# The reprotoxic adverse side effects of neurogenic and neuroprotective drugs: current use of human organoid modeling as a potential alternative to preclinical models

**DOI:** 10.3389/fphar.2024.1412188

**Published:** 2024-06-14

**Authors:** Mariam M. Abady, Ji-Seon Jeong, Ha-Jeong Kwon, Abdullah M. Assiri, Jongki Cho, Islam M. Saadeldin

**Affiliations:** ^1^ Organic Metrology Group, Division of Chemical and Material Metrology, Korea Research Institute of Standards and Science, Daejeon, Republic of Korea; ^2^ Department of Bio-Analytical Science, University of Science and Technology, Daejeon, Republic of Korea; ^3^ Department of Nutrition and Food Science, National Research Centre, Cairo, Egypt; ^4^ Deperament of Comparative Medicine, King Faisal Specialist Hospital and Research Centre, Riyadh, Saudi Arabia; ^5^ College of Veterinary Medicine and Research Institute for Veterinary Science, Seoul National University, Seoul, Republic of Korea

**Keywords:** drug-induced reprotoxicity, organoid model, neurotherapeutic drug, side effect, toxicicity

## Abstract

The management of neurological disorders heavily relies on neurotherapeutic drugs, but notable concerns exist regarding their possible negative effects on reproductive health. Traditional preclinical models often fail to accurately predict reprotoxicity, highlighting the need for more physiologically relevant systems. Organoid models represent a promising approach for concurrently studying neurotoxicity and reprotoxicity, providing insights into the complex interplay between neurotherapeutic drugs and reproductive systems. Herein, we have examined the molecular mechanisms underlying neurotherapeutic drug-induced reprotoxicity and discussed experimental findings from case studies. Additionally, we explore the utility of organoid models in elucidating the reproductive complications of neurodrug exposure. Have discussed the principles of organoid models, highlighting their ability to recapitulate neurodevelopmental processes and simulate drug-induced toxicity in a controlled environment. Challenges and future perspectives in the field have been addressed with a focus on advancing organoid technologies to improve reprotoxicity assessment and enhance drug safety screening. This review underscores the importance of organoid models in unraveling the complex relationship between neurotherapeutic drugs and reproductive health.

## 1 Introduction

**FIGURE 1:**
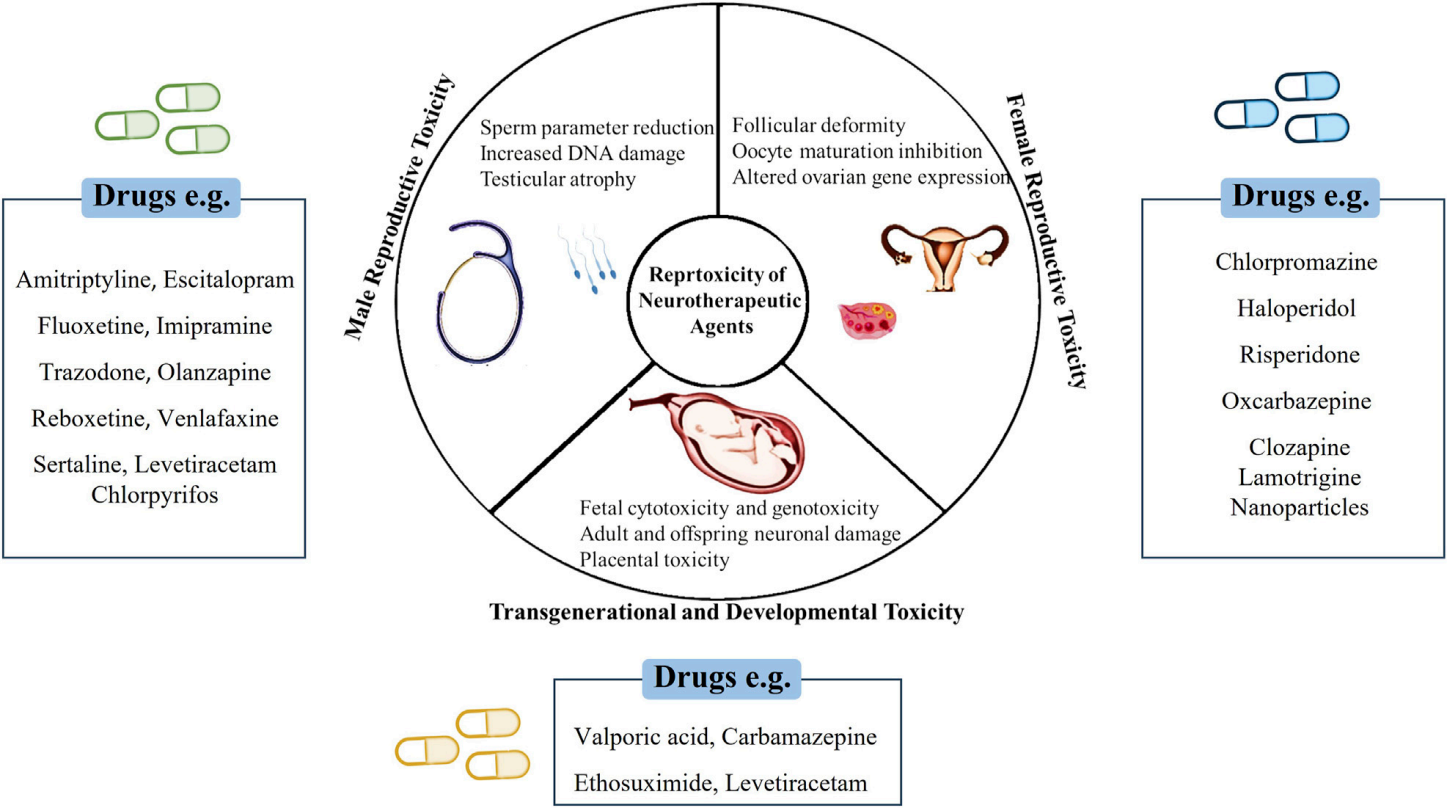
Schematic representation of neurotherapeutic agents’ impact on various reproductive health.

Drug-induced toxicity poses a significant challenge in drug research and development, often leading to failures in clinical trials and subsequent drug withdrawals (Vo et al., 2019). Reproductive toxicity, encompassing both reproductive and developmental toxicities, substantially contributes to drug withdrawal, accounting for approximately 3% and >10% of drug discontinuations and preclinical toxicology-related attrition, respectively (Siramshetty et al., 2016). To mitigate such risks, early assessment of the toxic properties of chemical compounds is paramount in drug development to mitigate such risks (Brannen et al., 2017). In reprotoxicity assessments, the impairment of male and female reproductive capacities and induction of nongenetic harmful effects on offspring are evaluated (Piparo, 2010) (Figure 1). However, traditional experiments for assessing chemical toxicity profiles, especially in animal models, are costly and time-consuming, with toxicity tests in animal models accounting for a significant proportion of compliance-related testing costs. Furthermore, the results of animal-based reprotoxicity tests may not always accurately predict human responses, adding complexity to toxicity endpoint assessments (Höfer et al., 2004). Additionally, discerning whether a compound directly affects reproduction or causes systemic toxicity that indirectly impacts reproductive systems further complicates the interpretation of experimental data. Consequently, the use of animal experiments alone might not fully reveal human responses to new drugs or provide reliable risk assessments, necessitating alternative approaches for toxicity assessment.

This review thoroughly examined neurotherapeutic drug-induced reprotoxicity in organoid modeling. The review first focused on stem cell applications for testing, exploring their potential and limitations. Then, it delved into the complex relationship between neurotherapeutic drugs and reproductive health, highlighting reprotoxic adverse effects. The second section offered a comprehensive analysis of the impact of neurotherapeutic drugs on reproductive health. Lastly, it showed future directions to improve technology fidelity and practical applications.

## 2 A brief history of organoids

Three-dimensional (3D) culture systems are generated using suspension culture to avoid direct contact with the plastic dish. This can be achieved through scaffold or scaffold-free methods. Scaffolds composed of biological or synthetic hydrogels mimic the natural extracellular matrix (ECM), and Matrigel^®^ is the most prevalent matrix. Matrigel, a complex protein mixture derived from Engelbreth–Holm–Swarm (EHS) mouse sarcoma cells, contains adhesive proteins such as collagen, entactin, laminin, and heparin sulfate proteoglycans, providing structural support and ECM cues to cells. In scaffold-free methods, cells are cultured in droplets of defined medium suspended from a plate by gravity and surface tension (Unbekandt and Davies, 2010). Alternatively, 3D organoid structures can be formed using the air–liquid interface method, in which cells are cultured on a basal layer of fibroblasts or Matrigel initially submerged in a medium. As the medium evaporates, the upper cell layers are exposed to air, promoting polarization and differentiation (Kalabis et al., 2012).

The idea of *in vitro* regeneration of organisms traces back to 1907, when Henry Van Peters Wilson demonstrated that dissociated sponge cells could self-organize and regenerate an entire organism (Wilson, 1907). Subsequently, experiments involving cell dissociation and reaggregation in the mid-20th century resulted in the generation of various organs from dissociated amphibian pronephros (Holtfreter, 1943) and chick embryos (Weiss and Taylor, 1960). In 1964, Malcolm Steinberg proposed the differential adhesion hypothesis, suggesting that cell sorting and rearrangement could be explained by thermodynamics mediated by varying surface adhesion (Locke, 2012). After the isolation and establishment of pluripotent stem cells (PSCs) from mouse embryos in 1981 (Evans, 1981; Martin, 1981) and human embryonic stem cells (ESCs) in 1998 (Thomson et al., 1998; Thomson et al., 1998), significant advancements were noted in stem cell research. Subsequently, induced pluripotent stem cells (iPSCs) were developed by reprogramming mouse and human fibroblasts, leading to a transformative impact on stem cell and organoid studies (Takahashi and Yamanaka, 2006; Takahashi et al., 2007; Yu et al., 2007).

**FIGURE 2:**
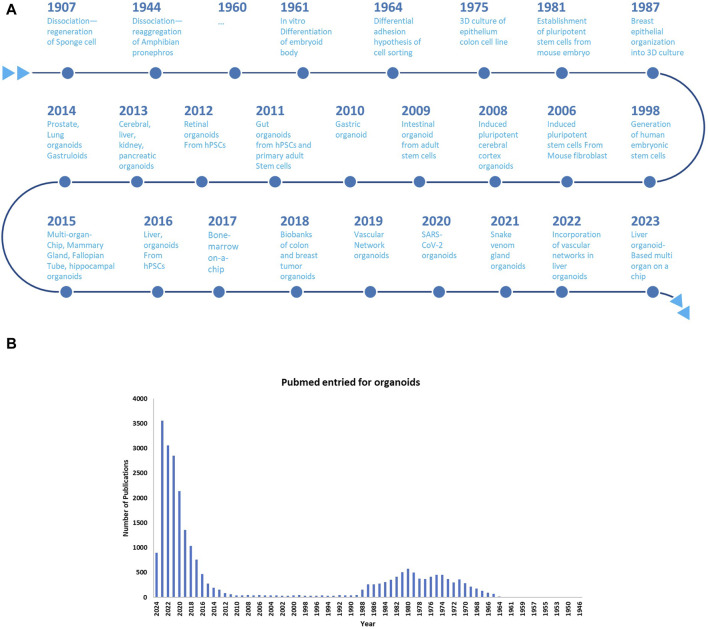
**(A)** Chronological evolution of three-dimensional (3D) organoid systems; **(B)** Increasing adoption of 3D Organoid Cultures in biological research: A PubMed Analysis till the year 2024.

In 1987, significant efforts were made in enhancing cell culture conditions to mimic the *in vivo* microenvironment. A previous study revealed that breast epithelial cells could generate 3D ducts and lumina when cultured on EHS ECM extract, enabling the production and secretion of milk protein; this phenomenon cannot be achieved in traditional two-dimensional (2D) culture (Li et al., 1987). Another study demonstrated that alveolar type II epithelial cells retain their specialized functions when cultured on ECM matrix, underscoring the crucial role of cell-matrix interactions in tissue homeostasis and differentiation (Shannon et al., 1987). The transition from 2D to 3D organoid culture was exemplified by the creation of cerebral cortex tissue from ESCs using the 3D aggregation culture technique (Eiraku et al., 2008). A groundbreaking study in 2009 demonstrated that adult intestinal stem cells expressing single leucine-rich repeat–containing G protein-coupled receptor five could form 3D intestinal organoids in Matrigel, organizing themselves into crypt–villus structures without requiring a mesenchymal niche (Sato et al., 2009) (Figure 2).

## 3 Organoid modeling techniques

Despite advancements in organogenesis research, fully replicating the human body *in vitro* remains challenging. Biological models like 2D/3D cell cultures and animal models have been established to mimic human physiology but face limitations (Shariati et al., 2021). Conversely, stem cell technology holds immense promise for unraveling disease mechanisms and offering potential therapeutic interventions through human tissue modeling. In 1998, the advent of human blastocyst-derived ESCs, which can differentiate into all three germ layers during gastrulation, marked a significant milestone in this field. These cells have been extensively utilized in laboratories globally. The groundbreaking establishment of iPSCs from adult human fibroblasts in 2007, which was achieved through the expression of four key transcription factors (SOX-2, KLF4, OCT3/4, and c-MYC), further expanded the repertoire of PSC sources. This breakthrough enabled the long-term culture of stem cells and establishment of *in vitro* 3D structures, laying the foundation for precision medicine. In addition to ESCs and iPSCs, mesenchymal stem cells (MSCs) are also crucial in translational research due to their immune modulation and regenerative abilities (Thomson et al., 1998; Takahashi et al., 2007; Ballini et al., 2017).

3D cell culture models offer a more accurate *in vitro* environment than 2D cultures. Organoids from primary tissue or stem cells hold potential in regenerative medicine and personalized medicine (Hynds and Giangreco, 2013). However, they vary based on cell type. Pluripotent stem cells mimic fetal tissue but may face heterogeneity issues. Adult stem cells are well-defined but challenging to isolate, limiting their potential (Forbes et al., 2018; Tuveson and Clevers, 2019).

### 3.1 Organoids for the male reproductive system

The male reproductive system is intricate, encompassing the testes, ducts, glands, and penis. The testes are vital for sperm and testosterone production, while ducts aid in sperm transport and maturation. Seminal vesicles, prostate, and penis are crucial for reproduction. Dysfunction can cause issues like infertility and cancer. Studying in humans is challenging, but *in vitro* models like primary cultures and tissue explants are used. 3D organoids show promise for research (Patrício et al., 2023).

#### 3.1.1 Testicular organoids

Testicular organoids, derived from healthy or diseased tissues, replicate testis structure and function. Baert et al. (2019) developed human testicular organoids by seeding adult and teen primary testicular cells into agar blocks, resulting in compact structures that can produce testosterone, form tight junctions, and support germ cell renewal. Other methods like alginate-based hydrogels and 3D bioprinting have also been used to generate testicular organoids, supporting spermatogenesis and Leydig cell functionality Matrigel®-based testicular organoid cultures in rodents, pioneered by Alves-Lopes et al., replicate *in vivo* testis features. They utilized a Matrigel gradient system for co-culturing Sertoli and germ cells. Typically, testicular organoid techniques involve direct cell-Matrigel mixing or a medium-Matrigel blend (Pendergraft et al., 2017; Yuan et al., 2020). In their study, a novel three-layer gradient system was developed by optimizing culture conditions, including the number of cells and concentration of Matrigel. Spherical–tubular structures were assessed using Sox9, Ddx4, and Scp3 as markers. Positive Ddx4 and Scp3 staining indicated the presence of germ cells, suggesting spermatogenesis potential. Sertoli cells, which are crucial for spermatogenesis, were positive for Scp3. Zo-1 expression suggested a potential blood–testis barrier (BTB), although this finding requires validation. Ki67 staining revealed the presence of proliferating cells (Wu et al., 2022).

These organoids exhibited a functional blood-testis barrier and maintained undifferentiated germ cells for prolonged periods (Alves-Lopes et al., 2017). Testicular organoids from porcine cells in collagen hydrogel mimic native testis architecture. 3D printing and microfluidic systems improve organoid fidelity (Vermeulen et al., 2019). Innovative techniques such as 3D printing and microfluidic systems have also been employed to enhance the fidelity of testicular organoids. Baert’s group utilized 3D-printed scaffolds to develop structurally compartmentalized organoids from mouse testicular cells, promoting tubulogenesis and supporting germ cell differentiation (Richer et al., 2021). Testicular organoids offer vast potential for studying male reproductive health, including spermatogenesis and hormone regulation. They provide avenues for disease modeling, drug testing, and personalized medicine in male infertility (Patrício et al., 2023).

Recently, Stopel et al. produced testis organoids from primary testicular cells of neonatal mice using transwell inserts. Our findings demonstrate that these organoids form tubule-like structures and exhibit cellular organization similar to that observed in live testicular tissue (Stopel et al., 2024).

#### 3.1.2 Epididymis organoids

The epididymis, which is vital for sperm maturation, is segmented into the caput, corpus, and cauda in larger mammals, with an additional initial segment in rodents (Pinel et al., 2019). It features a single convoluted tubule with a pseudostratified epithelium containing principal, clear, basal, and halo cells (Pinel et al., 2019). The blood-epididymis barrier (BEB) regulates luminal content and protects spermatozoa. Epididymal transit enhances sperm motility and fertilization potential through the luminal microenvironment and motility-affecting molecules (Sullivan and Belleannée, 2017). Generation of epididymal organoids was initially attempted using spheroid cultures from single cells, with human epididymal cells forming spheres under 2D conditions. Acinus formation in cultured rat epididymal basal cells was dependent on fibroblast growth factor (FGF) and dihydrotestosterone (Mandon et al., 2015). Basal cells could differentiate into principal cells, indicating existence of stem cell (Mandon et al., 2015). Recent developments by Leir et al. and Pinel and Cyr involved human and rat epididymal basal cells, respectively, forming 3D cultures (Leir et al., 2020; Pinel and Cyr, 2021).

#### 3.1.3 Organoids for prostate glands

The prostate gland, essential for sperm nourishment and transport, comprises luminal cells, basal cells, and rare neuroendocrine cells within a pseudostratified epithelium (Crowley and Shen, 2022). Prostatic fluid, containing zinc, citric acid, prostate-specific antigen (PSA), and choline, is pivotal for sperm liquefaction post-ejaculation, facilitated by PSA degrading Semenogelin I and II. Novel experimental models have emerged for studying prostate cancer (PCa) due to limitations in traditional cell lines and 2D cultures for drug screening. PCa organoids, derived from various cell sources, recapitulate the tumor microenvironment, aiding in understanding tumor development, progression, and therapy response. Examples include CRPC-derived organoids predicting enzalutamide sensitivity based on genetic alterations. High-throughput imaging assays enhance drug response analysis in diverse PCa phenotypes. Organoids elucidate mechanisms of drug resistance, like dual loss of TP53 and PTEN conferring resistance to anti-androgens. They serve in drug development and testing, using co-culture models to study microenvironmental effects and metastasis. Challenges include experimental variability and biopsy sample representativeness, with ongoing optimization for improved clinical outcomes in advanced PCa (Conteduca et al., 2020; Elbadawy et al., 2020; Gleave et al., 2020; Choo et al., 2021; Dhimolea et al., 2021).

Notably, urethral complications, from injury or congenital issues, present treatment challenges. Advances in stem cell research, notably 3D bioprinting, offer solutions. Tissue-engineered urethral grafts, pioneered by Atala and refined by Raya-Rivera, show promise in treating pediatric patients with urethral defects (ATALA et al., 1999; Raya-Rivera et al., 2011). Kajbafzadeh et al. explored regenerative methods, showcasing cell sheet techniques’ effectiveness in urethral reconstruction (Kajbafzadeh et al., 2017). Challenges persist in 3D bioprinting regarding implant mechanical strength and biocompatibility. Efforts in developing urethral organoids and cultivating corpus spongiosum structures concurrently hold promise for clinical advancements (Patrício et al., 2023).

### 3.2 Organoids for the female reproductive system

The female reproductive system comprises the ovaries, fallopian tubes, uterus, cervix, and vagina. This system responsible for gamet and sex hormone production and pregnancy. Female reproductive tissue organoids, including human endometrial organoids, effectively model endometrial physiology and pathology. Derived from diverse stem cell sources, they accurately mimic glandular structures and functions, responding to hormones and replicating conditions like endometriosis and cancer. Utilizing CD146+ mesenchymal stem cells for endometrial-like epithelium creation offers prospects for regenerative medicine and embryo implantation studies. Additionally, 3D stromal cell models enable research on decidualization and angiogenesis. Further exploration is needed to fully leverage endometrial organoids for understanding implantation challenges and early pregnancy failure (Hennes et al., 2019; Mittal et al., 2019; Zambuto et al., 2019; Cui et al., 2020; Wiwatpanit et al., 2020).

#### 3.2.1 Vulva organoids

The vulva, comprising various structures such as the labia majora, labia minora, and clitoris, serves as the initial defense barrier for the female reproductive tract. Although no organoids have been derived directly from the vulva, insights can be obtained from skin organoid studies due to the similarity in epithelial composition. Organoid studies on the skin have revealed spatiotemporal aspects of epidermal development and facilitated the long-term expansion of keratinocytes, offering a potential model for studying gene alterations implicated in vulvar diseases and carcinogenesis. Sweat glands, crucial for microbial homeostasis, have been studied using organoid cultures, suggesting a possible avenue for developing vulvar sweat gland-derived organoids. Overall, vulva-derived organoids hold promise for understanding epithelial biology, microbiome interactions, and diseases like genital infections and vulvar cancers (Boonekamp et al., 2019; Diao et al., 2019).

#### 3.2.2 Vaginal organoids

Research on vaginal development, primarily conducted in mice, has highlighted the intricate interplay between epithelial cells and the underlying stroma, influence of hormone receptor genes, and pivotal role of the Wnt/β-catenin pathway (Heremans et al., 2021). Recently, Ali et al. (2020) established a sophisticated 3D organoid culture system using mouse vaginal epithelial cells. This innovative model revealed the critical roles of Wnt and BMP signaling pathways in maintaining the stem cell niche within the vaginal epithelium. By meticulously controlling the culture conditions and manipulating key signaling molecules such as EGF, TGF-βR, and ROCK inhibitors, they expanded and sustained these organoids *in vitro* (Ali et al., 2020). Moreover, the identification of specific markers, such as AXIN2, provided insights into cellular hierarchy and lineage differentiation within the vaginal epithelium.

#### 3.2.3 Cervix organoids

The cervix, which is vulnerable to human papillomavirus -induced cancer, lacks accurate modeling in 2D cultures. 3D organoids provide a physiologically relevant platform for studying cervical cancer mechanisms. Cervical organoids, derived from patient biopsies, express specific markers and exhibit differentiation, offering insight into cervical cancer development. They enable studies on pathways like Wnt signaling in tumor progression (Heremans et al., 2021). Moreover, cervical organoids offer potential applications in personalized medicine, allowing for the testing of patient-specific drug responses. Maru et al. (2020) successfully developed cervical organoids from patient-derived biopsies using a specific medium containing RSPO1, Noggin, EGF, ROCKi, and Jagged-1. Cervical organoids exhibited enhanced expression of SCJ markers compared to traditional cell lines and demonstrated differentiation into both endo- and ectocervical cell types. Chumduri et al. (2021) also created long-lasting endocervical-like organoids from patient samples, reliant on Wnt agonists RSPO1 and WNT3A, showing potential differentiation towards ectocervical characteristics (Chumduri et al., 2021). Maru et al. (2019a, b) developed cervical clear cell carcinoma organoids using established culture conditions. Xenografting these organoids in mice allows for more clinically relevant treatment efficacy evaluation (Maru et al., 2019a; Maru et al., 2019b).

#### 3.2.4 Endometrial orgnaoids

The endometrium, which lines the uterus, sheds monthly under hormonal regulation. It consists of two layers: lamina basalis and lamina functionalis. The source of the endometrium remains controversial, and possible sources include stem and bone marrow-derived cells. Uncertainty exists regarding the hierarchy of proposed stem cell candidates and their translation to humans. Studies have been conducted to understand endometrial regeneration signaling. Previous 3D culture attempts were limited by short lifespans and inadequate *in vivo* mimicry, indicating the need for more representative models. For instance, Iguchi et al. (1985) successfully cultured luminal mouse endometrial cells on collagen gel matrices in serum-free conditions, exhibiting characteristics akin to adenogenesis, despite their short-lived nature (Iguchi et al., 1985). Similarly, Rinehart et al. (1988) seeded 3D endometrial glands on Matrigel-coated plates, resulting in structures with apicobasal polarity and preserved intercellular connections, albeit spreading out into 2D monolayer colonies (Rinehart Jr et al., 1988). Previous efforts faced challenges like low serum requirements, limited long-term maintenance, and incomplete endometrial characteristics. Ongoing work aims to develop advanced 3D endometrial models. Recently, organoids were cultured successfully using a defined medium (Boretto et al., 2017; Turco et al., 2017; Haider et al., 2019). RSPO1 or CHIR99021 activation of Wnt/β-catenin signaling was essential for their development, reflecting Wnt’s role in uterine gland formation. *In vivo* lineage tracing identified AXIN2+ cells as potential stem cell candidates in the mouse uterus (Syed et al., 2020). Human endometrial organoid development did not require exogenous WNT3A. Inhibition of BMP (Noggin) and TGF-β/Alk (A83-01) pathways was crucial, alongside EGF, FGF10, 17β-estradiol (E2), insulin (ITS), and inhibition of p38 MAPK, ROCK, and sirtuin. Hormone treatment replicated the menstrual cycle and early decidualization (Haider et al., 2019; Hennes et al., 2019; Bui et al., 2020; Cochrane et al., 2020; Syed et al., 2020). Trophoblast organoids were derived with minimal differences in medium. Marinić et al. (2020) derived endometrial gland organoids from the term placentas with slight modifications, showing hormone responsiveness and distinct molecular patterns (Marinić et al., 2020).

Importantly, adenomyosis and endometriosis involve ectopic endometrial tissue. Adenomyosis is within the uterine wall, while endometriosis involves tissue outside the uterus. Primate studies offer insights, but ethical concerns arise. Human-derived 3D organoids show promise for studying endometriosis (Heremans et al., 2021). Enriching organoid cultures with diverse cell types for studying adenomyosis and endometriosis is crucial. A reported co-culture model includes adenomyotic epithelial cells, stromal cells, and myocytes (Mehasseb et al., 2010). Although AXIN2+ cells were suggested as endometrial cancer (EC)-initiating cells in mice, the human counterpart remains unidentified (Syed et al., 2020). Patient-derived EC cell lines (e.g., Ishikawa, RL95-2) showed genomic stability but lacked intra-tumor heterogeneity (Van Nyen et al., 2018). Mouse xenograft models showed fair engraftment rates but struggled to replicate tumor microenvironments (Depreeuw et al., 2015). 3D culturing techniques, including spheroid cultures, revealed altered metabolism and drug susceptibility. Organoid development relied on key factors like RSPO1, EGF, and FGF2, with validated genomics showing mutations in ARID1A, CTNNB1, and PTEN (Boretto et al., 2019). Organoids were tested for sensitivity to chemotherapeutics and inhibitors targeting PI3K and mTOR pathways (Pauli et al., 2017; Heremans et al., 2021). Boretto et al. (2019) showed that organoids can replicate different endometrial states, including cancerous ones like Lynch syndrome mutations. This lays the groundwork for advanced organoid models for co-culture systems, drug testing, and gene-editing studies (Boretto et al., 2019).

#### 3.2.5 Fallopian tube organoids

Fallopian tube organoids derived from fallopian tube epithelial cells (FTECs) offer insights into infertility, tumor etiology, and drug effects (Chang et al., 2020). FTE organoids exhibit polarized columnar cells, tight junctions, and functional similarities to native tissue (Chang et al., 2020). They replicate mucosal fold architecture, express secretory markers, and respond to hormonal cues akin to the fallopian tube epithelium (Chang et al., 2020). These organoids present a valuable model for studying fallopian tube biology and pathology. Additionally, Fallopian tube organoids derived from FTECs or iPSCs accurately model fallopian tube biology and pathology, exhibiting distinct gene profiles and anatomical features compared to 2D cell lines. iPSC-derived organoids replicate fallopian tube anatomy through precise differentiation steps and expression of specific markers (Yucer et al., 2017). These organoids serve as disease models for conditions like chronic *chlamydia* infection (Kessler et al., 2019). However, challenges remain in long-term culture maintenance and achieving functional maturity for studying high-grade serous ovarian cancer (sHGSC) (Cui et al., 2020).

### 3.3 Embryonic and fetal organoids

Organoids have been developed from the embryonic and fetal stages. Trophoblast organoids derived from cytotrophoblasts (CTBs) model early placenta formation and placental diseases (Turco et al., 2018). Cultured in trophoblast organoid medium (TOM), CTB organoids closely mimic the morphology, differentiation ability, and gene expression patterns of human placental villi (Haider et al., 2018; Haider and Beristain, 2023). They demonstrate stemness, proliferation, and fusion characteristics akin to villous cytotrophoblasts (vCTBs), making them suitable for modeling implantation. While CTB organoids hold promise for disease modeling and investigating trophoblast invasion, further optimization is needed to enhance self-renewal, specificity, and differentiation (Turco et al., 2018). Additionally, human iPSCs produce trophoblast cystic structures resembling trophectoderm for implantation studies. Previous 3D models lacked true organoid characteristics (Wong et al., 2018; Cui et al., 2020).

Modeling the ovarian surface epithelium (OSE), the origin of most malignant ovarian tumors is crucial for exploring endometriosis etiology (Lawrenson et al., 2009). While 3D culturing of normal OSE remains unexplored for organoid construction, human oocytes have been mimicked in 3D structures derived from human embryonic stem cells (hESCs), showing meiotic entry and oocyte-like characteristics (Jung et al., 2017). These follicle-like cells (FLCs) express oocyte-specific markers, providing a platform for investigating human germ cell development and gene mechanisms, including noncoding RNAs (Jung et al., 2017; Cui et al., 2020).

Meanwhile, studying early human embryo development is challenging due to limited access to embryonic tissues. Pluripotent stem cells and surplus blastocysts offer insights, but ethical constraints restrict their use (Haider and Beristain, 2023). Recent advances in stem cell-based blastocyst models, known as blastoids, resembling natural blastocysts, provide insights into early embryonic development. They exhibit spatial organization similar to epiblast, hypoblast, and trophectoderm-like cells, with efficiencies ranging from 2% to 80%. Differentiation protocols prioritize lineage expansion, resulting in blastoids resembling blastocyst morphology and implantation processes (Theunissen et al., 2014; Rostovskaya et al., 2019). Blastoids aid in studying embryogenesis, implantation, and early pregnancy. Co-culture with endometrial cells reveals intercellular communication’s role in blastocyst attachment, with applications spanning developmental biology, fertility, and embryo safety (Kagawa et al., 2022).

Additionally, human placental research faces hurdles due to ethical constraints and limited tissue access. Trophoblasts, crucial for nutrient exchange, show species-specific developmental pathways, despite shared functions with rodents (Haider and Beristain, 2023). In 2018, 2D and 3D long-term regenerative trophoblast cultures were established from progenitor cytotrophoblasts (CTB) of first-trimester chorionic villi (<8 weeks gestation) (Haider et al., 2018; Okae et al., 2018). These cultures express trophoblast lineage genes, exhibit hypomethylation of the ELF5 promoter, and express chromosome 19 miRNA cluster micro RNAs. They can be perpetuated long-term with specific signaling conditions, fostering stem-like states and spontaneous fusion into hormone-producing multinucleated syncytiotrophoblast (SCT) in both 2D and 3D conditions. Removal of Wnt-activating factors prompts CTB differentiation into invasive extravillous trophoblasts (EVT). Trophoblast organoids represent a significant advancement in in vitro models, closely mirroring trophoblast cell lineage complexity observed *in vivo* (Haider et al., 2018). Single-cell transcriptomics confirms differentiation trajectories along extravillous and villous pathways (Shannon et al., 2022). Recent work addressed the limitations of blastoid models, including inverted syncytial structures, by utilizing suspension culture with gentle agitation. This approach produced organoids with properly oriented large syncytial structures that secrete high levels of SCT-associated factors (Yang et al., 2024). The choice of trophoblast progenitor source is crucial for trophoblast organoid design. While primary CTBs and TSC lines can both yield organoids, those from TSC lines may better resemble EVT progenitor-like cells transcriptionally and in surface marker expression (Sheridan et al., 2021). Moreover, TSC lines cultured in 2D or 3D exhibit detectable levels of Class I HLA-A/B, with 3D culture partially reducing their expression (Sheridan et al., 2021). Recent findings suggest that progenitors in hTSC line-derived organoids resemble a developmentally downstream state akin to column CTB (Shannon et al., 2022b). Though hTSC lines are favored for trophoblast studies, evaluating their merits and limitations is vital, particularly considering recent reports on their derivation from pluripotent stem cell (PSC) sources and induced TSCs (Karvas et al., 2022; Soncin et al., 2022; Tan et al., 2022). Trophoblast organoids aid in studying stem cell dynamics, highlighting YAP1 signaling’s importance via inhibition and CRISPR-Cas9-mediated knockout experiments (Meinhardt et al., 2020). Additionally, trophoblast organoids have elucidated the role of TGFβ signaling in EVT development, showing that exogenous TGFβ impulse is necessary for EVT marker expression, while its inhibition results in pro-migratory/pro-invasive features (Haider et al., 2022). Moreover, trophoblast organoids have been utilized to study vertical viral infection routes and ensuing inflammatory responses in decidual cells. Human cytomegalovirus infection triggers a Type III interferon response in trophoblast organoids, offering protection to decidual cells (Yang et al., 2022). iPSC-derived TSC organoids have also shed light on ZIKA and SARS-CoV-2 virus interactions with trophoblasts (Karvas et al., 2022). However, it is important to note limitations such as the inverted nature of the organoid and expression of MHC class I ligands not typically seen in progenitor CTB or SCT, which may impact interpretations related to pathogen-host modeling.

Interestingly, 3D organoids are crucial for modeling embryonic development, especially for the pituitary gland and hypothalamus (Chukwurah et al., 2019). The SFEBq method enables the three-dimensional culturing of ES cells, promoting differentiation into ectodermal derivatives (Watanabe et al., 2005; Eiraku et al., 2008; Sasai et al., 2012). Utilizing SFEBq cultures, hypothalamic neurons were successfully induced from mouse ES cells (Wataya et al., 2008). Growth factor-free, chemically defined medium (gfCDM) supplemented with Sonic Hedgehog (SHH) optimally induces hypothalamic neuron differentiation (Suga, 2016). Crucially, specifying the rostral hypothalamic fate of mouse ES cells relied on removing exogenous growth factors rather than adding specific inductive signals (Chukwurah et al., 2019).

## 4 Neurotherapeutic drugs and reproductive health

Neurotherapeutic drugs play critical roles in managing neurological disorders, and their impact on reproductive health has been increasingly recognized. As depicted in Table 1, these drugs exert diverse effects on various aspects of reproductive function, including sperm quality, hormone levels, and embryonic development. Understanding these interactions is essential for optimizing treatment strategies and minimizing potential adverse effects on reproductive outcomes.

**TABLE 1:** Neurotherapeutic agents’ adverse impacts on reproductive systems and fetal development.

Therapeutic class	Drug	Mechanisms	Example toxicities	Study type	References
Antidepressant	-Amitriptyline	-Formation of micronuclei	Mouse-derived spermatogonia and Spermatocyte	*In vitro*	Solek, et al. (2021)
	-Escitalopram	-Increase in telomeric binding factor (TRF1/TRF2) protein expression			
	-Fluoxetine	-Initiation of apoptotic cell death			
	-Imipramine	-Varied toxicity on mouse spermatogenic cells			
	-Trazodone	-Decreased sperm concentration, motility, and normal morphology; increased sperm DNA damage			
	-Olanzapine	-Elevated serum levels of FSH, LH, and testosterone			
	-Reboxetine	-Augmented oxidative stress			
	-Venlafaxine				
Selective serotonin reuptake inhibitors	Fluoxetine	Maladaptive offspring production	flea *Daphnia magna*	*In vivo*	Campos, et al. (2016)
	Sertaline	-Increased sperm DNA damage and induced histopathological lesions	Male rat		Atli, et al. (2017)
		-Abnormal sperm morphology and increased malondialdehyde (MDA) degeneration in cellular-tubular structures			
		-Elevated serum LH and testosterone levels			
		-Enhanced oxidative stress (OS)			
		Testicular toxicity			
	Citalopram	-Decrease in sperm motility	Men	Clinical	Safarinejad, 2008
	Escitalopram	-Abnormal sperm DNA fragmentation			Koyuncu, et al., 2011
	Fluoxetine				Safarinejad, 2008; Atli, et al., 2017
	Paroxetine				Tanrikut, et al., 2010
Antipsychotics	Chlorpromazine	-Increased activity of caspases-3, -8, and -9			Elmorsy, et al., 2017
	Haloperidol	-Elevated ROS (Reactive Oxygen Species) production	Female rat	*In vivo*	
	Risperidone	-Decreased total intracellular glutathione levels	Rat’s ovarian theca interstitial cells	*In vitro*	
	Clozapine	-Heightened lipid peroxidation (LPO)			
	Olanzapine	-Elevated prolactin levels	Man	Clinical	Konarzewska, et al., 2009
	Risperidone	-Reproductive hormone disorders identified			
		Sexual dysfunction observed			
Antiepileptics		-Initially upregulates Aldh1a2	Zebrafish embryo toxicity	*In vivo*	Beker van Woudenberg, et al., 2014
	-Valporic acid	-Subsequently downregulates Cyp26a1			
	-Carbamazepine	-Suggests a teratogenic mechanism			
	-Ethosuximide	- Hatching and Motor Activity			
	-Levetiracetam	-Pericardial Edema			
		-Motor Activity Suppression			
	Valporate	-Elevated serum concentrations of Testosterone	Women	Clinical	Morrell, et al., 2002; Asif, 2017
		-High prevalence of Menstrual Disorders, PCO, and PCOS			
		-Increased concentration of Androgens	Man		Mikkonen, et al., 2004; Asif, 2017
		-Abnormalities in sperm quality			
		-Reduced testicular volume			
	Oxcarbazepine	-Higher prevalence of PCO	Women		Mikkonen, et al., 2004; Asif, 2017
		-Higher serum concentrations of Androgens			
		-Higher serum concentrations of DHEAS			
		-Increased frequency of morphologically abnormal sperm	Man		Artama, et al., 2004; Asif, 2017
		-Lower serum testosterone levels			
	Carbamazepine	-Menstrual disorders linked to reduced bioactive E2 levels, indicated by altered E2/SHBG ratio	Women		Isojarvi (2001)
		-Reduced sperm concentration and high frequency of poorly motile sperm	Man		Perucca (2004)
		Induces hepatic P450 enzymes, elevating SHBG levels and lowering bioactive androgens			
	Lamotrigine	-High incidence of abortion and embryo lethality	Female rat	*In vivo*	Padmanabhan, et al., 2003; Hejazi and Taghdisi, 2019
		Congenital malformations and intrauterine growth retardation			
		-Decreased pup birth rate			
		-Significant reduction in body weight	Male rat		Daoud, et al., 2004
	Vigabatrin	-Reduction in the weight of testes, epididymis, seminal vesicles, ventral prostate, and vas deferens			
	Gabapentin				
	Levetiracetam	Dose-dependent decreases in sperm concentration, motility, and normal morphology	Male rat	*In vivo*	Baysal, et al. (2017)
		Increased sperm DNA damage observed			
		Alterations in oxidative stress markers indicating tissue damage			
Anti-cholinesterase	Dimethoate		Male Mice	*In vivo*	Verma and Mohanty, 2009
	Chlorpyrifos	-Reduced epididymal and testicular sperm counts			Joshi et al., 2007; Mor and Soreq, 2011
		-Decreased serum testosterone concentration			
		Pathological degeneration of seminiferous tubules			
		-Reduction in testicular glycogen and sialic acid content			
		-Increased cholesterol and protein content, dose-dependent			
	Malathion	-Increased testicular acid phosphatase activities			Choudhary et al., 2008; Mor and Soreq, 2011
		-Inhibition of testosterone secretion by Leydig cells			
Opioid	Methadone	-Considerable increase in oxidative stress levels	Male rats	*In vivo*	Haddadi, et al., 2020
	Buprenorphine	Loss of gonadotropin hormones observed			
		Changes detected in sperm parameters (Haddadi, Ai et al., 2020)			
	Morphine	-Testis atrophy observed			Bu, et al., 2011; Ghowsi and Yousofvand 2015; Moinaddini, et al., 2023
		Reduction in the number of germ cells			
		Weight loss in the testis, prostate, and seminal vesicles			
		Associated with morphine dependence			
Nanoparticles	Silver nitrate NPs	-Follicular growth deformities, oocyte maturation inhibition	Female rat	*In vivo*	Charehsaz, et al., 2016
		-Damaged neurons in hippocampal regions of adult and offspring rats			
	Titanium dioxide NPs	Concentration-dependent alteration in ovarian gene expressions	Female Mice	*In vivo*	Karimipour, et al. (2018)
	Aluminum oxide NPs	-Placental toxicities -Cytotoxicity and genotoxicity	*Ex vivo* Chinese hamster ovary cell line	*In vitro*	Di Virgilio, et al., 2010

### 4.1 Antidepressants

Depression affects approximately 300 million people worldwide and is a significant global health issue (Sołek et al., 2021b). Antidepressants, initially developed in the 1950s, constitute the primary treatment for depression, and they target neurotransmitter imbalances in the brain (Sartorius et al., 2007). However, long-term use of antidepressants, often necessary for full therapeutic benefits, can lead to sexual dysfunctions, affecting patients’ self-esteem and treatment compliance ((Montejo et al., 2019). These dysfunctions include reduced sexual desire, arousal difficulties, and orgasmic dysfunction (Higgins et al., 2010). The relationship between depression, pharmacotherapy, and sexual dysfunction is complex and lacks clear clinical guidelines (Hellstrom, 2008; Segraves and Balon, 2014). Antidepressants modulate neurotransmitters like serotonin, norepinephrine, and dopamine, influencing the sexual response cycle, and potentially explaining their effects on sexual function (Graf et al., 2019). Further research into direct interactions between antidepressants and reproductive cells is necessary to better understand and manage their side effects (ST et al., 2006; Solek et al., 2021).

#### 4.1.1 The reprotoxic side effects of treating depression

Most authors agree that Antidepressants and neuroleptic drugs are associated with sexual dysfunction, potentially due to their reproductive toxicity, impacting germ cell development, embryonic cell apoptosis, and fertility (Spiller et al., 2009; Kanatsu-Shinohara et al., 2010; Sato et al., 2011; Sato et al., 2012). One study demonstrated decreased ATP production in spermatogenic cells treated with amitriptyline hydrochloride, escitalopram, fluoxetine hydrochloride, imipramine hydrochloride, mirtazapine, olanzapine, reboxetine, and venlafaxine hydrochloride after 48 and 96 h, suggesting potential impairment of mitochondrial function (Sołek et al., 2021a).

Under stress, cells often arrest their cycle to facilitate repairing damage, resulting in changes to the cell cycle profile. Antidepressant-induced oxidative stress leads to increased DNA fragmentation and micronuclei formation, potentially due to reduced mitochondrial potential and irreversible damage (Aitken et al., 2014). Notably, antidepressants may influence the cell cycle, potentially inducing apoptotic cell death via various pathways. In particular, changes in cyclin D2 activity, governing the G1 to S phase transition, were observed, alongside the activation of proteins involved in cell cycle regulation (Biber et al., 2018). The activation of proteins (p16, p21, p27, p53) suggests their role in regulating the cell cycle via CDK inhibition (p16), DNA replication initiation (p21), G1/S phase transition (p27), and cell division (p53) (Roshdy and Fyiad, 2010; Mao et al., 2011; Battal et al., 2013; Biber et al., 2018). This activation hints at DNA damage repair and adaptive responses. Dysregulation of the cell cycle, crucial for spermatogenesis, may impair germ cell development, induce embryonic cell apoptosis, and reduce fertility (Spiller et al., 2009; Kanatsu-Shinohara et al., 2010; Sato et al., 2011; Sato et al., 2012). Antidepressants influence cell differentiation and proliferation through glucocorticoid receptor phosphorylation, upregulating p27 and p57, and induce cell cycle arrest in non-spermatogenic cells by inhibiting ERK1/2 kinase phosphorylation, altering gene expression in the p21/p53 pathway (Krishnan et al., 2008; Pechnick et al., 2011).

Disturbances in mitotic and meiotic processes, chromosomal aberrations, and reductions in sperm count, motility, and morphology are consistently observed across experimental sets (Alzahrani, 2012; Hassanane et al., 2012). Furthermore, the crucial protein NuMa, responsible for organizing and stabilizing the mitotic spindle apparatus, may be affected, leading to abnormal mitotic spindle formation after exposure to antidepressant drugs (Bhattacharya et al., 2013). Additionally, the interaction of cytotoxic drugs with tubulin subunits may disrupt microtubule polymerization and depolymerization, thereby impairing the function of the mitotic spindle. These highlight the complex impact of antidepressant treatment on reproductive health. Telomere dysfunction leads to genomic instability, apoptosis, or cellular senescence (Picco et al., 2016). Research has indicated a relationship between the levels of TRF1 and TRF2 proteins, expression of p53 and MAPK kinase, and the induction of apoptosis. Cell lines treated with antidepressants showed increased TRF1 and TRF2 synthesis. While some studies provide evidence of telomere length reduction in depressive disorder patients (Ridout et al., 2016), and changes in the amount of mitochondrial DNA (mtDNA) or telomere length due to stress and depression (Powell et al., 2018), there is insufficient data on TRF1 and TRF2 expression changes after antidepressant treatment.

In the final phase, cell death mechanisms are activated, marked by increased cleavage of caspase three and reduced synthesis of Bcl-2. The interplay of proteins within the Bcl-2 family significantly influences cell fate, determining whether cells survive or undergo apoptosis (Djordjevic et al., 2012). Additionally, the Bcl-2 family mediates the intrinsic pathway of apoptosis, wherein mitochondria play a significant role. Some findings suggest that patients with depressive disorders exhibit low expression of anti-apoptotic Bcl-2, which is increased with antidepressant treatment (Sakr et al., 2013; Khaksar et al., 2017). Preclinical studies have also demonstrated that antidepressants elevate Bcl-2 levels, offering protection against apoptotic cell death through interaction with mitochondrial voltage-dependent anion channels. Apoptosis is common in the testis as a safeguard mechanism for eliminating defective germ cells (Shukla et al., 2012) which is exacerbated by antidepressant usage inducing DNA fragmentation and lipid peroxidation, leading to cellular damage (Atli et al., 2017b). While confirmation in complex *in vivo* models is necessary due to testicular tissue complexity, these findings offer valuable insights into antidepressant-induced reproductive toxicity (Mytych et al., 2017).

#### 4.1.2 Selective serotonin reuptake inhibitors

Selective serotonin reuptake inhibitors (SSRIs), commonly used to treat depression, raised concerns for endocrine-disruption. Human studies suggest developmental toxicity, reversible sexual dysfunction, and sperm DNA damage. In aquatic environments, fluoxetine, an SSRI component, acts as a neuroendocrine disruptor, affecting fish fertility and behavior. *Daphnia magna’* studies reveal SSRIs alter reproductive responses and offspring size, reversed by cyproheptadine. These findings highlight serotonin’s role in reproductive regulation, necessitating further research (Campos et al., 2016).

In arthropods, serotonin neurons regulate vital processes like oogenesis, growth, and behavior. Yet, understanding of non-decapod crustaceans like Daphnia is limited. Evidence suggests serotonergic neurons influence growth and reproduction in Daphnia, inferred from SSRIs’ effects and the presence of serotonin biosynthesis enzymes in their genome. Neurotoxin 5,7-dihydroxytryptamine (5,7-DHT) damages serotonergic neurons, leading to oxidative damage, hypoxia, and neurotoxicity (Lamichhane et al., 2014; Campos et al., 2016; Nation Sr, 2022).

Daphnia’s stress response involves adjusting reproductive investment to maximize fitness. Serotonergic interneurons in the brain regulate this process, as evidenced by SSRIs’ effects. Fluoxetine increases serotonin-immunoreactivity under low food conditions, mimicking “optimum” reproductive conditions, while 5,7-DHT reduces it, aligning with reduced reproduction. These findings highlight serotonin’s crucial role in Daphnia’s reproductive investment regulation, revealing adaptive mechanisms in varied food environments (Gorbi et al., 2011; Gaukler et al., 2015).

Infertility affects 15% of couples, with male factors contributing to 30%–50% of cases. Factors like varicocele, infections, endocrine disorders, obesity, radiation, and drug use, including SSRIs, can impact male fertility by affecting sperm parameters and hormonal balance (Atli et al., 2017a).

SSRIs like sertraline, fluoxetine, and trazodone can impact male fertility due to their effects on sperm parameters and hormonal balance. SSRIs commonly prescribed for depression, such as sertraline, fluoxetine, and trazodone, are associated with sexual side effects, potentially affecting sperm count and morphology. For instance, Trazodone an antidepressant with anxiolytic and sleep-inducing effects, is widely used for insomnia. While clinical studies have linked SSRIs to decreased sperm quality, trazodone’s reproductive toxicity remains underexplored, particularly in males of reproductive age (Nørr et al., 2016). A study found that TRZ administration reduced sperm concentration in male rats, motility, and normal morphology while increasing sperm DNA damage and testicular degeneration. Elevated serum levels of FSH, LH, and testosterone, along with oxidative stress in testicular tissue, were also observed (Ilgın et al., 2018). These hormonal changes were associated with decreased sperm quality and testicular degeneration. Additionally, trazodone exposure led to oxidative stress, as indicated by elevated levels of malondialdehyde (MDA) in testicular tissue, reflecting lipid peroxidation (Rahal et al., 2014; Sabeti et al., 2016; Ilgın et al., 2018). Clinical research on patients undergoing TRZ treatment is crucial for identifying potential reproductive toxicity, emphasizing the importance of monitoring sperm parameters before, during, and after TRZ therapy.

#### 4.1.3 Lithium

Lithium salts are commonly used to treat major depressive disorders, with exposure occurring through various sources such as drinking water, food, and the environment. While low levels can alleviate depression, prolonged therapeutic doses may lead to complications affecting the renal, nervous, thyroid, and circulatory systems. Furthermore, Li + exposure can result in teratogenic effects and sterility (Aral and Vecchio-Sadus, 2008; Ommati et al., 2021).

As evidence, One study aimed to explore Li^+^'s adverse effects on testicular tissue, spermatogenesis, and hormones using *in vitro* and *in vivo* models. *In vitro*, Leydig cells were cultured with Li^+^ at escalating concentrations (0–100 ppm), while mice were given Li+ in drinking water (0–100 ppm) for 5 weeks *in vivo* (Ommati et al., 2021). Testicular and sperm samples were analyzed. Notably, oxidative stress has been implicated in testis and sperm injury in animals treated with Li^+^. Li^+^ affects testosterone biosynthesis *in vivo* and *in vitro*. Importantly, mitochondrial impairment plays a critical role in sperm and Leydig cells abnormality, and testis injury with Li^+^-exposure (Folgerø et al., 1993). Li^+^ induces decreased sperm motility via mitochondrial impairment and reduced ATP levels (Yousefsani et al., 2020). Li^+^ adversely affects the reproductive system, with oxidative stress playing a crucial role. Sperm mitochondria are particularly vulnerable, leading to reduced motility. Additionally, Leydig cell ATP levels decrease, impacting testosterone synthesis and secretion and increased lactate dehydrogenase release. These effects may impair reproductive function in long-term Li + treatment. Dysfunction in sperm mitochondria may result from disrupted membrane potential and electron transport chain interference (Filippa and Mohamed, 2019; Yousefsani et al., 2020), as well as increasing mitochondrial permeability and facilitating the release of various cell death mediators into the cytoplasm. Recently, safe and clinically applicable agents like amino acids and peptides enhance mitochondrial function and energy metabolism. (Ben Saad et al., 2017; Jamshidzadeh et al., 2017; Heidari et al., 2019). Further studies are warranted to elucidate the precise mechanisms underlying Li^+^-induced reproductive organ injury, potential interactions of adjunctive treatments with Li^+^'s pharmacological effects, and the translation of experimental findings into clinical practice.

### 4.2 Antipsychotics (Aps)

Individuals with psychosis often require lifelong treatment with AP, which can lead to various side effects, including abnormal movements, weight gain, diabetes, and reproductive disorders like menstrual irregularities or amenorrhea in women (Elmorsy et al., 2017a). Despite past beliefs that typical APs were more toxic than atypical ones, research suggests that both types show similar reproductive toxicity (Murke et al., 2011).

Notably, APs can lead to reproductive disorders through hyperprolactinemia, primarily induced by dopamine D2 receptor inhibition. While typical antipsychotics are often associated with hyperprolactinemia (about 57% of patients), atypical ones generally do not affect prolactin levels except for risperidone (Wong, 2007). However, there’s no direct correlation between prolactin levels and menstrual irregularities (Lee and Kim, 2006). Regardless of hyperprolactinemia status, antipsychotics are linked to similar rates of reproductive dysfunction and may reduce peri-ovulatory estradiol levels (Canuso et al., 2002). Hence, while hyperprolactinemia is significant, it may not fully explain antipsychotic-induced reproductive toxicity.

APs induce cytotoxic effects in rat ovarian theca interstitial cells by inhibiting mitochondrial bioenergetics. Both *in vitro* (Elmorsy et al., 2014) and *in vivo* (Martins et al., 2008) studies demonstrate that APs induce oxidative stress in non-reproductive cells (Elmorsy et al., 2017a; Elmorsy et al., 2017b).

The study explores how antipsychotics (APs) induce reproductive toxicity via oxidative stress in rat ovarian theca interstitial cells (TICs). APs cause cell damage, increased caspase activity, and oxidative stress (high ROS production, reduced glutathione levels, and heightened lipid peroxidation). Antioxidants alleviate this damage, suggesting a potential therapeutic approach, but clinical research is needed for future validation (Elmorsy et al., 2017a).

Schizophrenia, a worldwide condition marked by symptoms like hallucinations and confusion, often starts during the reproductive years, affecting brain neuroendocrine functions and disrupting reproductive processes (Ardıç et al., 2021). Antipsychotic medications used for schizophrenia treatment can cause reproductive toxicity by affecting hormonal regulation, leading to sexual dysfunction, disrupted spermatogenesis, and abnormalities in epididymal maturation (Solomon et al., 2019; Zhao et al., 2019). Olanzapine (OLZ), a second-generation antipsychotic used for schizophrenia and bipolar disorder treatment, affects various neurotransmitter systems. It acts on multiple neurotransmitter receptors, including dopamine, serotonin, muscarinic, adrenergic, and histaminergic receptors. It can increase prolactin levels in females, leading to adverse effects like amenorrhea, impotence, and sexual dysfunction (Fernandes et al., 2019; Meftah et al., 2020). Elevated prolactin levels can cause hypogonadism hindering sperm production and causing issues like delayed spermatogenesis, reduced semen quality, and abnormal testicular tissue in both humans and animals (Akram et al., 2019; Zhao et al., 2019). Research on male rats showed that OLZ administration reduced normal sperm morphology and caused toxicity in testicular tissue, attributed to increased oxidative stress, Leydig cell damage, and disruption of hormone regulation (Ardıç et al., 2021). Of particular importance, Elevated ROS levels in 25% of infertile males contribute to sperm defects and dysfunction, affecting sperm functions like capacitation, acrosome reaction, mitochondrial sheath stability, and motility (Sikka and Hellstrom, 2017). Sperm cells’ susceptibility to ROS is attributed to their high levels of unsaturated fatty acids in the membrane and limited cytoplasmic ROS-neutralizing enzymes (Sidorkiewicz et al., 2017). Lipid oxidation can lead to compromised cell membrane integrity, heightened membrane permeability, enzyme inactivation, DNA impairment, and ultimately, cell apoptosis (Sikka and Hellstrom, 2017), potentially leading to decreased sperm count, activity, motility, and abnormal sperm morphology (Sidorkiewicz et al., 2017). Following olanzapine treatment, testicular GSH levels decreased notably in addition to a significant increase in SOD activity in the olanzapine-treated group (Ardıç et al., 2021). Subsequently, free radicals induce sperm oxidative stress, impairing function and fertility. Elevated ROS levels in the testes lead to semen oxidative stress, associated with idiopathic infertility. OLZ reduces GSH levels, indicating oxidative stress, while increased SOD levels suggest a rapid ROS response. High doses of OLZ exacerbate oxidative stress, impacting sperm morphology and testicular structure (Simon and Carrell, 2013; Elghaffar et al., 2016). More research needed to understand OLZ’s reproductive toxicity. Monitoring sperm and hormone levels in patients is essential for risk assessment (Ardıç et al., 2021).

### 4.3 Antiepileptics

Epilepsy, affecting 20–40 million people worldwide, is characterized by abnormal neuronal activity and is managed with long-term medication using antiepileptic drugs (AEDs), tailored to individual needs (Asif, 2017). While newer AEDs offer improved tolerability, challenges such as adverse effects and drug interactions persist (Fisher et al., 2005). Certain AEDs may impact reproductive health, contributing to reproductive disorders (Isojärvi et al., 2004). The pharmacological landscape of AEDs has expanded with advancements in drug design and insights into seizure mechanisms (Mohanraj and Brodie, 2003). Epilepsy management requires a comprehensive approach that addresses therapeutic gaps and minimizes adverse effects (Kwan and Sander, 2004). Reproductive dysfunction, including reduced fertility in both sexes, is prevalent among epileptic patients, possibly due to epilepsy itself or antiepileptic medication (Artama et al., 2004). Catamenial epilepsy, influenced by hormonal changes, exhibits estrogen-induced seizures and progesterone’s anticonvulsant effects (Isojärvi et al., 2004). Thyroid hormones also affect seizure activity, with thyrotoxicosis possibly increasing seizure risk (Asif, 2017). Epilepsy correlates with various reproductive disorders, including irregular menstrual cycles and decreased potency which are exacerbated by untreated epilepsy (Asif, 2017). Seizures and interictal periods disrupt hormone release, affecting reproductive function. AEDs may also impact reproductive hormones, causing menstrual disorders, reduced potency, and diminished sexual interest (Asif, 2017). Limited data exist on newer AEDs like Oxcarbazepine and their reproductive effects.

Women with idiopathic generalized epilepsy (IGE) have higher rates of reproductive disorders like polycystic ovaries (PCO), hirsutism (HA), and polycystic ovary syndrome (PCOS) compared to those with localization-related epilepsy (LRE) or without epilepsy (Morrell et al., 2002). IGE is linked to anovulatory cycles, polycystic appearing ovaries, elevated BMI, and HA. In contrast, LRE is associated with PCOS related to left-sided focus and hypothalamic amenorrhea and hyposexuality linked to right-sided focus (Asif, 2017). Epilepsy disrupts pituitary hormone regulation, with left-sided focus epilepsy linked to disturbances in the temporo-limbic hypothalamic-pituitary axis, with left-sided focus epilepsy showing increased LH secretion and LH/FSH ratio (Herzog et al., 2003). Prenatal genetic factors may influence epilepsy and hormone regulation, potentially creating a bidirectional relationship with reproductive disorders (Asif, 2017).

#### 4.3.1 Valproic acid

In women with epilepsy (WWE), valproic acid (VPA) use predicts reproductive disorders like polycystic ovary syndrome (PCOS), hirsutism (HA), and polycystic ovaries (PCO). Starting VPA at a younger age correlates with increased HA and PCOS incidence, while obesity does not significantly predict reproductive issues (Asif, 2017). Regression analysis helped isolate these factors’ effects, consistent with previous research reports (Morrell et al., 2002; Isojärvi et al., 2004). Further research is needed to confirm and explore factors influencing reproductive health in WWE, as few studies have utilized regression analysis to identify contributing factors (Morrell et al., 2002; Herzog et al., 2003; Mikkonen et al., 2004).

WWE had comparable fertility rates to controls, but lower fertility if epilepsy persisted into adulthood. MWE also experienced reduced fertility with active epilepsy in adulthood. Few population-based studies have explored epilepsy’s impact on fertility despite its correlation with reproductive disorders (Artama et al., 2004). Active epilepsy and medication during adulthood were associated with reduced fertility in WWE, while remission before adulthood led to similar fertility rates as controls (Mikkonen et al., 2004).

Furthermore, Girish et al. (2014) examined sodium valproate-induced reproductive toxicity in male rats (Girish et al., 2014). Treatment at 400 mg/kg/day led to reduced body and testis weights, decreased sperm count and motility, and histological changes in the testes, including necrosis, atrophy in seminiferous tubules, and impaired spermatogenesis, as well as a notable reduction in Johnsen’s testicular score (Bairy et al., 2010; Cansu, 2010; Girish et al., 2014). Notably, sodium valproate disrupts cellular mechanisms, potentially inducing free radical formation and lipid peroxidation, which may disrupt testicular structure and function (Tamber and Mountz, 2012). Additionally, it promotes apoptosis in human and rat granulosa cells by increasing caspase-3 activity (Cansu, 2010).

Turning to developmental toxicity caused by VPA, it induces extensive transcriptional changes, with downregulated genes associated with RNA processing and chromatin modification/histone acetylation, consistent with its known histone deacetylase (HDAC) inhibitory action. It is classified as a developmental toxicology chemical, notably down-regulating mRNA expression of neuronal markers like NF-68, NF-200, NMDA-receptor, and GABAA-receptor. Moreover, therapeutic plasma concentrations in adults and children upregulate nestin mRNA expression, suggesting glial cell activation or neural precursor cell proliferation in response to neuronal cell death (Krug et al., 2013).

#### 4.3.2 Pregabalin

Pregabalin, six times more potent than Gabapentin, is widely used in psychiatry and neurology for treating epilepsy, anxiety, fibromyalgia, and neuropathic pain in diabetic patients. Acting on voltage-gated calcium channels, it provides analgesic and anxiolytic effects, also influencing the dopaminergic reward system. Despite misuse potential, its prescribed use is escalating (Shokry et al., 2020). Yet, when given at therapeutic doses to non-abusers, its abuse potential may be lower compared to benzodiazepines, stimulants, or opioids (Loftus and Wright, 2014). Pregabalin does not enhance euphoria or sexual ability; it may cause dysfunction, especially in epilepsy patients. Tramadol is highly toxic, affecting seminiferous tubules and sperm severely. Morphine, hashish, and heroin also impair sperm quality, with morphine’s effect on count and motility debated (Isojärvi et al., 2005). For instance, a study found that pregabalin negatively impacted male rat reproductive function, reducing serum testosterone levels without affecting pituitary gonadotropins. Histopathological examination revealed significant degenerative changes in the seminiferous epithelium and decreased cell counts for all spermatogenesis cells, Sertoli cells, and Leydig cells. Immunohistochemical analysis indicated increased apoptosis, shown by caspase3 expression (Shokry et al., 2020). Interestingly, Pregabalin toxicity may involve central action, inhibiting the hypothalamic-pituitary-gonadal axis. It decreases melatonin levels, reducing Leydig cell protection against oxidative stress and diminishing testosterone release. Additionally, its impact on serotonin leads to reduced thyroid hormone levels, further impairing Leydig cell development. Oxidative stress, implicated in apoptosis induction, plays a significant role in pregabalin toxicity (Zhao and Wang, 2012).

In female rats, pregabalin reduced pituitary gonadotropins but increased E2, progesterone, and testosterone levels. Ovarian histopathology showed increased atretic follicles and heightened apoptosis, consistent with barbiturates inducing ovarian atrophy. Tawfeeq et al. observed dose-dependent LH, FSH, and prolactin inhibition with pregabalin inhibition, correlating with elevated atretic ovarian follicles (Tawfeeq et al., 2016). Pregabalin’s toxicity affects the pituitary gland, stimulating E2 secretion or inhibiting progesterone’s effect on hypothalamic GnRH. Other drugs, like tramadol, induce ovarian failure, while oxymorphone partially inhibits ovulation (Shuey et al., 2008; El-Ghawet, 2015; Shokry et al., 2020).

#### 4.3.3 Anticonvulsant drugs

Anticonvulsant drugs like phenobarbital (PBT), phenytoin (PHT), and carbamazepine (CBZ) are associated with teratogenic effects when used during pregnancy, leading to major malformations, microcephaly, growth retardation, and minor facial and finger abnormalities in infants (Isojarvi, 2001; Perucca, 2004). The risk of congenital malformations is doubled in children born to mothers taking AEDs, with specific syndromes like fetal hydantoin syndrome (associated with PHT) and spina bifida (linked to VPA) highlighting these risks. Balancing the need to minimize AED exposure against controlling maternal seizures is crucial during pregnancy, with close monitoring recommended for pregnant women on AEDs (Wagh et al., 2011; Asif, 2017).

While first-generation AEDs like VPA and CBZ are known for teratogenic effects, limited data exist for newer compounds like ethosuximide (ETH) and levetiracetam (LEV)) (Pennell et al., 2012). ETH has shown teratogenicity and cognitive impairment in rodents, while LEV has demonstrated developmental effects only at very high doses (Mawhinney et al., 2013). Recent findings suggest LEV may be a safer alternative to VPA, with a lower risk of major congenital malformations in women with epilepsy of childbearing age.

A previous report investigated Zebrafish as a valuable model for assessing developmental toxicity, offering advantages for medium/high-throughput screening as an embryonic/larval model. A study aimed to enhance the predictability of the zebrafish model by employing an integrated screening strategy with various endpoints, including morphology, behavior, histopathology, kinetics, and phenotyping through *in situ* hybridization. Four AEDs (VPA, CBZ, ETH, and LEV) were selected as model compounds (Beker van Woudenberg et al., 2014). Histopathological analysis of VPA-treated larvae revealed reduced brain cellularity, particularly in the optic regions, highlighting histopathology as a sensitive endpoint. Motor activity analysis confirmed VPA’s neurodevelopmental toxicity, supported by literature evidence. CBZ primarily affected hatching and motor activity at lower concentrations, aligning with rodent and human studies indicating CBZ-induced neurodevelopmental disorders. ETH-induced pericardial edema and neurodegeneration, with biphasic dose-response effects observed. LEV exhibited significant motor activity effects at lower concentrations, suggesting its developmental neurotoxic potency, despite minimal structural damage (Kultima et al., 2010; Beker van Woudenberg et al., 2014).

Additionally, expression patterns of Aldh1a2 and Cyp26a1 indicated a link between gene expression and apical endpoints, suggesting the potential use of molecular markers for phenotype prediction (Menegola et al., 2006; Beker van Woudenberg et al., 2014).

Recent studies suggest LEV may harm sperm quality in rats, causing reduced concentration, motility, abnormal morphology, DNA damage, and testicular tissue damage in male rats via oxidative stress and hormonal imbalances. Human studies are needed to assess LEV’s reproductive risks and fertility impact. Clinical research should focus on reproductive toxicity and fertility in LEV-treated individuals, evaluating sperm DNA damage and oxidative status (Baysal et al., 2017).

### 4.4 Cholinergic toxicity

Various factors like toxic agents, malnutrition, illness, or stress can disrupt male reproductive function, impairing fertility via androgen production, germ cell development, and somatic cell maintenance. Cholinergic signaling proteins, vital for sperm function, are expressed in male reproductive tissues. Dysfunction, possibly induced by agricultural agents or stress, can harm fertility. Chemotherapeutic drugs may also affect cholinergic proteins, underscoring the need to understand cholinergic toxicity in male fertility (Mor and Soreq, 2011).

Spermatogenesis in testicular seminiferous tubules involves Leydig cells producing testosterone and Sertoli cells maintaining the microenvironment. It progresses through stages from spermatogonia to spermatozoa. Cholinergic innervation aids sperm transport and regulates production via acetylcholine, acting on receptors in smooth muscles. Nicotinic receptors are in parasympathetic ganglia, and muscarinic receptors are in smooth muscles, with acetylcholinesterase terminating signaling (Mor and Soreq, 2011).

Nicotinic acetylcholine receptors (nAChRs) in sperm aid fertilization and regulate motility, influenced by testicular proteins. nAChRs are involved in regulating Leydig cell function and testosterone secretion (Kumar and Meizel, 2005). Muscarinic acetylcholine receptors (mAChRs) in epithelial cells of sperm and Sertoli cells affect proliferation and luminal fluid composition (Avellar et al., 2010).

AChE has diverse isoforms like AChE-R and N-AChE, differing in their C- and N-domains due to alternative splicing and promoter usage. AChE-R is found in human and mouse sperm and is linked with differentiation by containing pseudointron I4, interacts with cellular proteins, and correlates with sperm motility. N-AChE, found in round spermatids’ acrosome, is involved in spermatogenic differentiation (Mor and Soreq, 2011). Exposure to anti-cholinesterase pesticides like malathion, dimethoate, and chlorpyrifos has been linked to testicular toxicity in rodents, affecting sperm counts, testosterone levels, and testicular histology, possibly through disruption of cholinergic signaling in Leydig cell function (Joshi et al., 2007; Choudhary et al., 2008; Ruchna Verma and Banalata Mohanty, 2009).

### 4.5 Molindone-induced reproductive toxicity

Molindone hydrochloride, a dopamine D2 and serotonin 5-HT2B receptor antagonist, is under investigation for treating impulsive aggression (IA). Developed for schizophrenia, it is now being studied as SPN-810, an extended-release version, for IA in attention-deficit/hyperactivity disorder. Rat studies reported CNS signs and prolactin increases. While developmental toxicity is not evident, its effects on fertility are unclear. Dopamine antagonism-induced prolactin elevation may affect reproduction differently among species, necessitating further evaluation, particularly in rats, for accurate human risk assessment (Krishna et al., 2017).

Regulatory-compliant DART studies on molindone HCl, a dopamine D2 receptor antagonist, found no teratogenicity or adverse fetal effects in rats (up to 40 mg/kg/day) and rabbits (up to 15 mg/kg/day) during organogenesis. These doses, with exposure margins of 69X and 6X over clinical levels, were well-tolerated (Gopalakrishnan et al., 2018).

Furthermore, a postnatal development study revealed no effects on survival, developmental milestones, or functional evaluations in rats. Transient reductions in pup weight gain were observed at the highest dose, but no post-weaning effects or impact on litter size. Maternal hypoactivity is possibly linked to a slight reduction in pup survival. Molindone HCl induced CNS-related signs and maternal toxicity in rats and rabbits, consistent with its pharmacology. Fertility studies in rats showed altered estrous cycle duration but no effects on mating or reproductive parameters in males or females (Gopalakrishnan et al., 2018). These effects, attributed to dopamine D2 receptor antagonism and prolactin secretion, are not considered relevant to human reproduction. Clinical trials support the reproductive safety of molindone HCl in target patients (Stocks et al., 2012; Gopalakrishnan et al., 2018).

### 4.6 Nanoparticles (NPs) and drug delivery nanocarriers

Nanotechnology in nervous system imaging and drug delivery offers precise treatment but raises concerns about nanoparticles’ (NPs) health risks (Pinheiro et al., 2021; Faiz et al., 2022; Jo et al., 2022; Qiao et al., 2023). NPs from various products may accumulate in tissues, posing risks to pulmonary, liver, kidney, and neurological issues. Their ability to breach barriers like the blood-brain barrier and the placenta raises concerns about reproductive health and fetal development (Hersh et al., 2022). In reproductive medicine, Gold nanoparticles aid cell visualization in ovarian carcinoma, and tocotrienol nanosized emulsions treat breast and ovarian tumors. NP exposure can affect sperm count, morphology, hormonal levels, and sexual behavior in males, and ovarian function in females (Ahmad, 2022). Public awareness about NP toxicity is crucial for both genders’ reproductive health and fetal development. NPs can disrupt the female reproductive system, governed by hormones, potentially leading to fetal abnormalities. Studies indicate acute and chronic toxic effects on reproductive tissues, highlighting concerns (Souza et al., 2021). NPs can penetrate biological barriers like the placenta, affecting male and female reproductive tissues. Zebrafish models offer insights into embryonic development due to ethical constraints with traditional animal models (Blum et al., 2012). NPs can disrupt reproductive function, leading to developmental and fertility issues. Research shows adverse effects on maternal weight, placental health, implantation rates, and hormone levels, impacting pregnancy outcomes (Brohi et al., 2017). In pregnant mice, cadmium oxide NPs delayed maternal weight gain and impacted placental weight, possibly affecting implantation. Silica and titanium oxide NPs reduced uterine weight and increased fetal reabsorption rates, suggesting adverse effects on reproductive tissues and fertility (Hou and Zhu, 2017).

#### 4.6.1 Effects of gold and titanium dioxide NPs on the male reproductive system

Male reproductive health is affected by NPs at molecular, cellular, and histological levels, requiring thorough toxicity evaluations (Habas et al., 2021). Titanium dioxide NPs during mouse pregnancy altered male offspring’s neurological tissues and affected Leydig and Sertoli cells, impacting reproductive growth (Umezawa et al., 2012). Intra-tracheal carbon-based NP exposure during pregnancy induces histopathological changes in seminiferous tubules, affecting sperm production in male offspring. Multi-walled carbon nanotube exposure causes reversible testicular damage without affecting fertility in mice (Iftikhar et al., 2021). Testicular nanoparticle accumulation impacts germ cell numbers, histopathology, and sperm motility. Water-soluble NPs have fewer toxic effects, while fat-soluble ones can induce apoptosis or inflammation, compromising male fertility (Hong et al., 2016).

Gold NPs in semen impaired sperm motility, while polyvinyl alcohol-coated iron oxide nanoparticles had no effect (Vassal et al., 2021). *In vitro* models using mouse and bovine sperm showed nanoparticle cytotoxicity, with silver nanoparticles most toxic (Mohammadinejad et al., 2019). Airborne nanoparticulate pollutants from industry pose reproductive health risks, evident in germ-line mutational changes in mice exposed to such pollutants (Jaishankar et al., 2014).

#### 4.6.2 Effects of lead and zinc NPs on the male reproductive system

Lead, a highly toxic heavy metal, is widespread in the environment due to human activities and poses significant health risks with neurotoxic and immunotoxic effects (Mahmood et al., 2012). Zinc oxide nanoparticles, valued for their biocompatibility, are used in products like sunscreens but can penetrate cells, exhibiting toxicity influenced by size and dosage (Keerthana and Kumar, 2020; Deore et al., 2021).

In research, the detrimental impacts of lead and zinc oxide nanoparticles on experimental animals’ reproductive organs have been elucidated, with outcomes dependent on dosage and exposure duration (Lee et al., 2016). Exposure to zinc oxide nanoparticles has been associated with notable declines in sperm counts and motility, likely due to induced oxidative stress (Lee et al., 2016). Earlier research has also highlighted toxic effects on the testis and epididymis from both zinc oxide nanoparticles and lead. (Deore et al., 2021). Indeed, oxidative stress emerges as a pivotal factor in metal-induced toxicity, marked by an imbalance between free radicals and antioxidants such as superoxide dismutase, catalase, and glutathione peroxidase (Ighodaro and Akinloye, 2018). This imbalance can precipitate organ toxicity and is implicated in various health conditions. Increased oxidative stress has been observed in the male reproductive systems following exposure to lead and zinc oxide NPs, underscoring their involvement in the toxic effects of these metals (Ighodaro and Akinloye, 2018; Deore et al., 2021).

#### 4.6.3 Effects of NPs on the female reproductive system

NPs’ toxic effects include female reproductive disruption, teratogenicity, and prenatal development issues, as they translocate to reproductive and fetal tissues through inhalation, ingestion, or dermal absorption (Dugershaw et al., 2020). Titanium dioxide (TiO_2_) NPs disrupted granulosa cell hormonal secretions, reducing pregnancy rates and altering ovarian gene expression in mice (Chen et al., 2003). Exposure to zinc oxide NPs during pregnancy or lactation poses health risks to mothers and embryos (Clementino et al., 2021). Nanoparticles accumulate in ovarian tissues in a size-dependent manner, with larger particles accumulating more than smaller ones (Raj et al., 2017). NPs affect oogenesis based on factors like size, surface charges, and exposure routes. Metals, metallic oxides, carbon-based NPs, and quantum dots penetrate female germline cells, inducing reactive oxygen species, DNA damage, and inflammation. Gold and silver NPs, including their alloys, accumulate in oocytes and cumulus cells, with silver NPs showing higher toxicity (Mao et al., 2022).

NPs have adverse effects on the female hormonal system by disrupting the hypothalamus-pituitary-ovarian axis, leading to neuro-hormonal instabilities (Hou and Zhu, 2017). Nickel NPs in rats disrupt hormones and damage ovaries, while titanium dioxide NPs affect hormone levels and follicles in female rats (Karimipour et al., 2018). Furthermore, quantum dots and calcium phosphate NPs disrupt ovarian cell activities and steroid synthesis pathways. NPs crossing cellular barriers impact placental function and fetal development, influenced by barrier thickness. Evolving placental barriers regulate substance exchange between maternal and fetal compartments (Woods et al., 2018). Understanding NP-biological barrier interactions is crucial for safer nanoparticle therapies (Herrick and Bordoni, 2019). Drug-delivering NPs can induce developmental toxicities, affecting fetal cell growth, differentiation, and genetic expression. Crossing placental barriers causes neurodevelopmental anomalies and DNA damage. *In utero* gene editing with multifunctional nanoparticles offers therapeutic promise with no fetal developmental adverse effects (Ricciardi et al., 2018). However, NPs may cause fetal abnormalities, with variations in toxicity among different types. Some, like amorphous silica-based NPs, exhibit no prenatal toxicities, while others, such as molybdenum-based NPs, impact maternal weight, fetal growth, and genetic stability (Mohamed et al., 2020).

Regulatory frameworks for nanoparticle reproductive toxicity need integration. Harmonized guidelines are essential for assessing risks to reproductive health from nanoparticles, requiring comprehensive data for clinical safety assessment (Grillo et al., 2021). Reproductive toxicity studies may be needed in later clinical trial phases based on product and patient considerations (Husain et al., 2015).

### 4.7 Opioids

Opium use, prevalent during events like the SARS-CoV-2 pandemic, poses risks to cognition and reproductive health (Khosravi, 2022). Morphine, its main alkaloid, disrupts hormonal balance via testicular opioid receptors, impairing sperm production and quality. Morphine induces ROS, damaging cell membranes and causing DNA fragmentation. Long-term use leads to dependence by affecting brain receptors, affecting key structures like the amygdala and hippocampus, and testicular opioid receptors, disrupting hormonal balance (Moinaddini et al., 2023). Replacement therapies like methadone and buprenorphine manage dependence. Methadone aids detox but can cause side effects, while buprenorphine, favored since 2001, has minimal placental transfer in pregnant women (Meyer et al., 2015; Robin et al., 2022; Moinaddini et al., 2023).

A study investigated sperm and testis parameters in morphine-dependent animals and those undergoing detoxification with methadone/buprenorphine (Moinaddini et al., 2023). Morphine affects testicular opioid receptors, impacting germ and glandular cells, increasing DNA fragmentation, and potentially causing infertility. Morphine use and detox affect mitochondrial activity and sperm motility; while improved viability is seen with methadone detox. Testicular weight and dimensions decrease with morphine use and detox (Moinaddini et al., 2023).

Chronic opium use in male rats alters testicular architecture, inducing inflammation and toxicity. Opium addiction reduces sexual activity via decreased testosterone levels, potentially causing sexual suppression and infertility. Hypophysial gonadal secretion function decreases, impacting sperm quality, which may result from direct action on gonads or via the hypothalamic-hypophysial-gonadal axis, affecting proper spermatogenesis and male sexual responses. Suppression of this axis results in reduced sperm count, semen quality, erectile function, and infertility. Further research is needed to understand opium’s reproductive health effects (Hejazian et al., 2007; Amin, 2013).

## 5 Examining neurotherapeutic drug-induced reprotoxicity via organoid modeling: Steps toward personalized therapy

There is a significant gap in the literature regarding the assessment of neurotherapeutic drug-induced reprotoxicity using organoid modeling. This limitation underscores the crucial need to develop and use reproductive organoids to study the toxic effects of neurotherapeutic drugs on reproduction. Moreover, it highlights the importance of incorporating organoid modeling into research methodologies rather than conventional approaches based on cell lines and mice to develop more clinically relevant and predictive models.

Antidepressants may affect semen parameters and male fertility, with mirtazapine potentially exerting fewer adverse effects on germ cell DNA damage than amitriptyline. However, studies based on germ cell lines may have limited human relevance. Further research on the effects of antidepressants on semen quality and fertility is crucial. Testicular organoids offer a promising solution for reducing animal use in toxicity studies, thereby addressing limitations in current clinical indicators for testicular toxicity. These 3D structures mimic the intricate cell interactions in the testes, providing a valuable tool for modeling normal development and pathophysiology and performing drug testing. Thus, testicular organoids hold immense potential for assessing reproductive toxicity while minimizing animal use and associated costs.

Wu et al. (2022) established an enhanced testicular organoid model comprising rat testicular cell homogenates to evaluate the reproductive toxicity of antidepressants, focusing on mimicking drug effects on various aspects of spermatogenesis and elucidating underlying mechanisms. Using such a model, amitriptyline and mirtazapine were selected, the two most commonly used antidepressants, to assess their impact on spermatogenic cells (Wu et al., 2022).

Testicular organoids were used to examine the effects of antidepressants. Compared with mirtazapine, amitriptyline induced greater apoptosis in the organoids with higher cell death rates. Amitriptyline exhibited greater cytotoxicity across cell lines than mirtazapine, indicating its higher potential for testicular damage (Wu et al., 2022).

Immunostaining confirmed the presence of germ cells and Sertoli cells, suggesting spermatogenesis initiation and BTB formation (Yuan et al., 2020). In the abovementioned study, both amitriptyline and mirtazapine exerted dose-dependent effects on spermatogenesis-related gene expression, with amitriptyline inducing greater toxicitythan mirtazapine (Yuan et al., 2020). Extended mirtazapine treatment suggested potential damage to undifferentiated spermatogonia (Yuan et al., 2020). At lower doses, none of the drugs affected Zo1 expression, preserving BTB integrity, whereas amitriptyline downregulated Zo1 expression at higher doses, indicating BTB damage (Yuan et al., 2020). Sertoli cells displayed resistance to drug-induced damage, particularly for mirtazapine (Wu et al., 2022). These findings underscore differing reproductive toxicity profiles of amitriptyline and mirtazapine, highlighting the need to assess drug effects on testicular function and spermatogenesis.

The organoid platform evaluates antidepressant toxicity on spermatogonia, with amitriptyline showing more significant effects on spermatogenesis genes than mirtazapine. Comparisons with mouse cell lines support these findings, validating the organoid model’s relevance. Despite refinement needs, the model is a valuable tool for drug toxicity screening and mechanistic studies on spermatogenesis, offering high repeatability and ease of operation. Pioneering research on rat testicular organoids may be translated to human models, elucidating clinically relevant drug reproductive toxicity (Wu et al., 2022).

Furthermore, lamotrigine, a commonly used drug for treating various neurological conditions, such as epilepsy and bipolar disorder, is associated with potential side effects, including adverse effects on reproductive health such as disrupted menstrual cycles, hormonal imbalances, and alterations in fertility parameters. Furthermore, emerging evidence suggests that lamotrigine can exert toxic effects on reproductive organs, particularly the endometrium (Ann et al., 2023; Mwangi et al., 2023; Rezk et al., 2024).

## 6 Advancements and challenges in assessing developmental toxicity

Human organoids, although promising, encounter challenges such as inconsistent batch outcomes and incomplete maturity, affecting their reproducibility and ability to replicate native organ functionality. Addressing the limitations of batch variability and heterogeneity through standardized protocols could enhance the reliability and applicability of human organoids (Velasco, Kedaigle et al., 2019). Additionally, efforts to augment organoid complexity involve integrating vascular and immune components to better mimic native tissue structures and functions (Hofer and Lutolf, 2021). Engineering techniques create vascularized and immune organoids, like brain organoids with microglia-like cells (Popova et al., 2021). Additionally, Biomaterials and microfluidic systems mimic *in vivo* cellular environments within organoids (Li et al., 2022).

**FIGURE 3:**
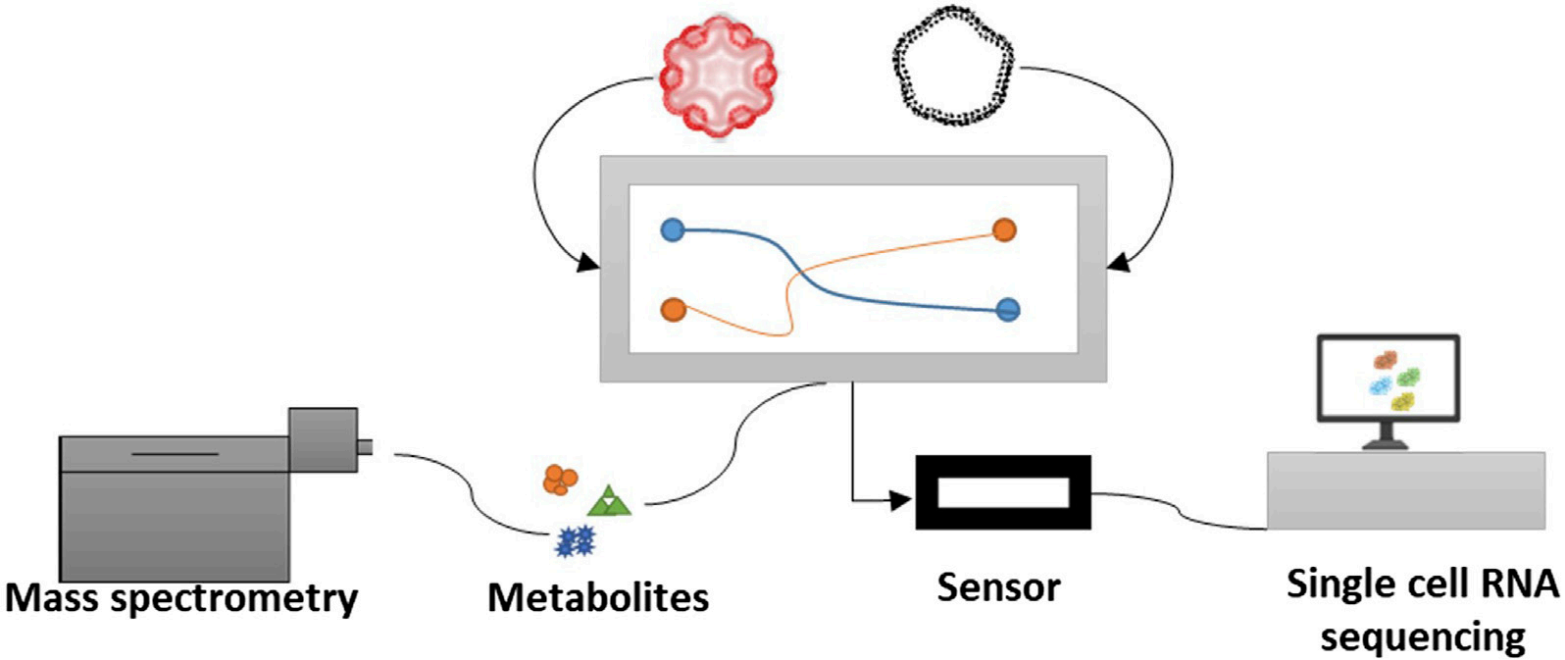
Integrated Multi-Organoid System on Chip: This model combines micro-engineered organoids (e.g., brain and reproductive organs) on chips with single-cell RNA sequencing (sc-RNA-seq) and mass spectrometry to investigate the systemic toxicity of neurotherapeutic drugs. Therapeutic compounds are introduced into the model to target brain diseases and assess their effects on reproductive organoids.

Another notable advancement is the emergence of “organs-on-chips” (OoCs) platforms, which faithfully replicate the dynamic microenvironment of human organs, facilitating *in vitro* assessment of systemic toxicity (Figure 3). OoC devices replicate tissue functions, aiding in understanding physiological dynamics and multi-organ connectivity. Mathematical modeling quantifies responses, while lab-on-a-chip platforms integrate microfluidic chips for dynamic cultures. In biosciences, *in silico* and theoretical modeling refine systems, with MoC systems modeling toxin processes for systemic toxicity insights. (Devall et al., 2021; Sung, 2022). For instance, embedding JEG3 on a chip can interact precisely with HUVECs, resembling the placenta unit in maternal-fetal interface studies (Mittal et al., 2019; Cui et al., 2020). Moreover, high-throughput single-cell RNA sequencing (scRNA-seq) has been instrumental in characterizing the transcriptome of individual cells within organoids, allowing researchers to identify alterations induced by toxic exposures. For example, scRNA-seq has been used to study the effects of prenatal exposure to toxic substances on cell development and differentiation (Wang et al., 2021). Despite its promise, the integration of scRNA-seq into developmental toxicity assessment with organoids remains limited (Devall et al., 2021).

Ferasyi et al. demonstrated the complexity of reproductive signaling pathways with a male reproductive axis model, highlighting hormone interactions among the central nervous system, pituitary gland, and gonads. Mathematical modeling elucidates fluid dynamics, with lumped-parameter models resembling electrical circuits and hydrodynamic models analyzing biofluid flows directly using the Navier-Stokes equation (Mazumdar, 2015; Ferasyi et al., 2016). Furthermore, pharmacokinetic (PK) and pharmacodynamic (PD) models predict compound effects on the endocrine system. PB PK-PD models, using ODEs, integrate signaling processes with mathematical relationships like Hill and Michaelis-Menten equations. Systems biology-based algorithms simulate network models, aiding drug screening in OoCs (Schnell, 2014).

Turning to microfluidics, it Microfluidics aids MEMS device design for biomedicine, handling nanoliter volumes and integrating sensors (Karolak et al., 2018; Manz et al., 2020). Laminar flow, influenced by shear stress, governs fluid behavior, with diffusion ensuring stable gradients. Computational fluid dynamics predict behavior, while tissue-mimicking chips study molecular events (Morshed and Dutta, 2018; Piemonte et al., 2018; Manz et al., 2020; Sung, 2022). Interestingly, multi-organ systems mimic interactions via microfluidic connections, aided by PBPK-PBPD models. Scaling optimizes design for predicting responses (Lee et al., 2017; Prantil-Baun et al., 2018).

Turning our attention to the reproductive tract, it is complex, housing gonads and vital organs like the ovary. Microfluidic biochips mimic ovulation, providing insights into infertility pathways. OoC devices integrate endocrine loops, aiding the study of endocrine-disrupting chemicals (EDCs)-triggered pathways with *in silico* methods (Xiao et al., 2017; Bodke and Burdette, 2021).

A novel endometrium-on-a-chip device simulated cyclic estradiol hormone effects on stromal and endothelial cells. It featured dual-chamber microfluidics with a porous membrane for co-culturing, maintaining steroid sensitivity for biochemical analysis. Endocrine organ-on-chip systems by Nguyen et al. and Gnecco et al. could benefit from integration with *in silico* algorithms or mathematical models, as shown by Lee et al. in a pancreas-muscle-liver OoC (Gnecco et al., 2017; Lee et al., 2017; Nguyen et al., 2017).

OoCs replicate dynamic hormone signaling in microfluidic environments, mimicking human reproductive pathways (Nawroth et al., 2018). Xiao et al. (Xiao et al., 2017) conducted a study combining microfluidic culture of the human reproductive tract with mathematical PK simulation. Their system orchestrated synchronized fluid flows to emulate the menstrual cycle hormone profile. Ovarian follicles generate hormones regulating downstream tissues, with an ODE system modeling inter-organ hormonal signaling for drug discovery and toxicological studies (Sung, 2022).

Additionally, the integration of omics technologies enhances the understanding of developmental toxicity mechanisms and facilitates biomarker discovery (Mao et al., 2024). Integration of mass spectrometry with omics technologies and organoid models enhances assay precision, refining compound potency ranking in reprotoxicity tests. Mass spectrometry provides insights into developmental toxicity mechanisms, identifying biomolecules, metabolic pathways, and biomarker signatures in organoids exposed to toxic compounds. This integration improves sensitivity and specificity, aiding in early detection and prediction of adverse developmental outcomes (Abady et al., 2023; Xu and Yang, 2024). This synergistic approach advances our ability to evaluate and mitigate developmental hazards in pharmaceutical and environmental contexts.

More research is needed to enhance reproductive disease modeling. The epididymis is pivotal for sperm maturation, but our understanding is limited. Few 3D epididymal organoids exist, with challenges including the blood-epididymis barrier’s integrity for drug testing. Additionally, mimicking the different portions of the epididymis remains a significant challenge, highlighting the need for further research in this area (Leir et al., 2020; Pinel and Cyr, 2021; Patrício et al., 2023).

Emerging research focuses on embryoids, organized embryo-like structures aiming to model integrated early embryonic development. Unlike organoids, they offer reproducible cellular organization and architecture within a shorter timeframe (Fu et al., 2021). Gastruloids, for example, serve as models of gastrulating embryos, providing insights into anterior-posterior axial patterning and potential teratogenicity assessment (Papalia et al., 2007; Mantziou et al., 2021). Saadeldin et al. (2024) conducted an experiment involving the co-cultivation of endometrial organoids (EOs) with embryos and made several significant observations. Notably, it was observed a five-fold increase in the cell number of co-cultured embryos and a seven-fold increase in the proportion of trophoblast outgrowths compared to control embryos. Additionally, embryos cultured with an EO-conditioned medium demonstrated a higher rate of attachment compared to other models, and remarkably, embryonic elongation was observed for the first time, providing a valuable tool for investigating the intricate processes involved in porcine embryo implantation (Saadeldin et al., 2024). These advancements show promise for toxicological studies, yet further research is needed to optimize their fine development and application (Papalia et al., 2007; Li et al., 2022).

## 7 Conclusion

The integration of advanced technologies such as organoids offers a promising approach to assess the reproductive toxicity of neurotherapeutic drugs. Organoids provide a physiologically relevant model that bridges the gap between traditional *in vitro* cell cultures and *in vivo* animal studies, allowing for more accurate assessments of drug effects on reproductive health. Despite their potential, current studies on neurotherapeutic drug-induced reproductive toxicity in organoid models are limited, highlighting the need for further research in this area. Addressing the current limitations of organoid technology, such as variability and maturity levels, is essential for understanding their full potential for toxicity screening and mechanistic studies. By advancing our understanding of the complex interactions between neurotherapeutic drugs and reproductive health, organoid modeling can lead to improved clinical management and reproductive risk mitigation strategies in drug development.
